# Improving the evidence base on multimorbidities through better research: a commentary on the U.S. HHS initiative, *Multiple Chronic Conditions: A Strategic Framework*

**Published:** 2013-12-24

**Authors:** William A. Satariano, Cynthia M. Boyd

**Affiliations:** ^1^School of Public Health, University of California-Berkeley, Berkeley, CA, USA; ^2^Division of Geriatric Medicine and Gerontology, Department of Medicine, Johns Hopkins University School of Medicine, Baltimore, MD, USA

**Keywords:** multiple chronic conditions, multimorbidity, population disparities, strategic framework, U.S. Department of Health and Human Services

“Multiple Chronic Conditions: A Strategic Framework” is a seminal report and the heart of a US strategic initiative, released by the U.S. Department of Health and Human Services (HHS) in December 2010, to focus the attention and resources of the US government on the research, practice, and policy implications of multiple chronic conditions (MCCs) [1]. The specific purpose of the report is “to catalyze change within the context of how chronic illnesses are addressed in the United States – from an approach focused on individual chronic diseases to one that uses a multiple chronic condition approach” [1]. The report observes that this process represents “a culture change, or paradigm shift, and the subsequent implementation of these strategies that will provide a foundation for realizing the vision of optimal health and quality of life for individuals with multiple chronic conditions” [1].

The issue of MCCs is of increasing relevance to the international community, as highlighted by a growing international literature over the past decade (see [2]). Thus, we posit that we can all learn from a conversation that builds a truly international perspective, recognizing that different countries have unique populations and health systems, to address the common theme of the health of people living with MCCs. The purpose of this special issue of the *Journal of Comorbidity* is to provide an international forum for consideration and discussion of the MCC report [1], as summarized in an “informed overview” prepared by Anand K. Parekh and Richard A. Goodman, from the HHS Office of the Assistant Secretary for Health (Parekh and Goodman) and National Center for Chronic Disease Prevention and Health Promotion, Centers for Disease Control and Prevention (Goodman) [3]. Both Parekh and Goodman were instrumental in the development of the MCC report [1]; and are, therefore, uniquely qualified to place the report in its historical context, recalling the important steps in its development. Parekh and Goodman also describe the overall goals of the strategic initiative, with a particular focus on the goal of facilitating research to fill knowledge gaps about MCCs and to establish an evidence base of interventions and systems that benefit individuals with MCCs [3]. For this research goal, they highlight the four specific research objectives. They provide an update of current steps by the HHS to enhance: (a) the external validity of clinical trials for people with MCCs; (b) the epidemiology of MCCs; (c) clinical, community, and patient-centered health research; and (d) research on disparities in MCCs within populations. Finally, Parekh and Goodman conclude by suggesting additional potential priorities for research in MCCs [3].

An international group of scholars was invited to react to the Parekh and Goodman summary of the MCC report [3]. Each scholar was asked to address one of the four research objectives, and, whenever possible, to highlight some of the current research, practice, and policy from their home countries. They were also encouraged, in particular, to focus on the “how” of these four core research topics: what they thought of the strategies in the framework; what can be learned from related strategies in other countries; the specific operational tasks that will help to accomplish these strategies; and finally, what the opportunities are to leverage resources to advance this agenda through international cooperation.

We thank our team of scholars for their excellent contributions. The scholars and their topics are given below.

Martin Fortin and Susan M. Smith – *Improving the external validity of clinical trials: the case of multiple chronic conditions*. Fortin and Smith are affiliated with the Université de Sherbrooke in Quebec, Canada, and the Royal College of Surgeons in Dublin, Ireland, respectively. Fortin and Smith agree with Parekh and Goodman [3] that individuals with MCCs are often excluded from clinical trials, generally considered to be the gold standard for the clinical and community-based research. As such, the generalizability or external validity of clinical trials is typically sacrificed for the internal validity of those trials. This, in turn, severely limits the development of an effective evidence base for clinical strategies to address MCCs. They emphasize that we must not only consider patient factors (the primary focus of their piece and the MCC report [1]), but that system factors are also relevant. Fortin and Smith contend that it is necessary to consider alternative and complementary designs for trials, alternative sources of evidence, including the development and use of sophisticated post-marketing surveillance, treatment fidelity studies, and prospective cohort studies. Finally, they agree that the innovative use of secondary data should be employed.

Francois G. Schellevis – *Epidemiology of multiple chronic conditions: an international perspective*. Schellevis, affiliated with the Netherlands Institute for Health Services in Utrecht, provides an international context for epidemiological data on MCCs using data from the USA and many other countries, and considers the underlying reasons for observed differences. He makes a strong case for the utility of focused epidemiological research to better inform the development of studies to evaluate the effectiveness of interventions in order to establish an evidence base to care for individuals with MCCs. Like other members of the international group, he agrees with the MCC report about the potential utility of secondary data analysis, especially regarding opportunities for cross-country comparisons. Schellevis also argues forcefully for the need to further examine the diversity among subpopulations within and between countries as a foundation to move forward and focus primary attention on intervention research.

Jose M. Valderas – *Increasing clinical, community, and patient-centered health research for preventing and managing multimorbidity*. Valderas, affiliated with the Universities of Exeter and Oxford in the UK, argues that this is the broadest of the four research objectives and describes a critical need to establish an evidence base to develop a range of effective patient-centered programs to improve clinical and community care for individuals with MCCs. He points out that these types of research are not distinct, and are by definition inter-related when done well. He highlights the focus on determining outcome priorities for people with MCCs. All of this work, argues Valderas, merits a refinement of an expanded conceptual model for the delivery of healthcare to individuals with MCCs.

Efrat Shadmi – *Disparities in multiple chronic conditions within populations*. Shadmi, from the University of Haifa, Israel, strongly agrees with the MCC report in that it is important to address the topic of disparities in incidence and management of MCCs, and that prevention is a fundamental mechanism to consider. While most research on MCCs focuses on an aging population, Shadmi contends that research on MCCs, especially among specific subpopulations, should provide new insights into health disparities by race, ethnicity, and socioeconomic status among adults in general. She also argues, however, that this research depends on the development of more sophisticated measures of MCCs to include acute conditions and general symptoms. With a more sophisticated evidence base, Shadmi believes that programs and policies can be developed to meet the special needs of diverse groups within and among populations.

We believe that the articles in this special issue make a compelling case that the study of MCCs, while challenging, will lead to a new generation of clinical and community-based programs and policies to enhance the health and well-being of a growing and increasingly diverse population. The MCC strategic initiative [1], as summarized so well by Parekh and Goodman [3], is both a report of what we know and what we need to know in this area. It includes an examination of work to date, including the early studies of Alvin Feinstein and his students in the 1970s and 1980s [4, 5]. The report, which we believe represents the most comprehensive and extensive to date, builds on previous programs, initiatives, and conferences from the USA and elsewhere [6–8]. It demonstrates a clear recognition of the promise of the field, and provides a specific directive for action to move research, practice, and policy forward. The reactions to the MCC initiative [1], as expressed by Fortin and Smith, Schellevis, Valderas, and Shadmi, indicate that this enthusiasm, while tempered by a full appreciation of the difficulties and challenges in this area, is shared by others in countries outside the USA. We believe that the MCC initiative is an important first step. In the end, the MCC initiative will be evaluated by the extent to which the goals are met. In the near term, however, the process and progress of the initiative will be assessed in part by whether the financial and funding commitments to MCC research are sufficient to achieve meaningful results that impact the other goals, and whether the USA actively collaborates with other countries to meet these important research objectives and leverage finite resources.

Finally, in addition to the topics raised in this rich discussion, we recommend three additional areas for consideration.

First, attention to living with MCCs must take a life-course view, as many of the risk factors for MCCs date to childhood, and perhaps even gestation, and this view may be particularly relevant as we address disparities. This includes the risk factors for particular comorbid conditions among people with specific index conditions. Altering this course will take community and environmental strategies as well as those focused on individuals or health systems, and the evaluation of such programs may stretch well beyond a particular political cycle and typical funding mechanisms.

Second, research should focus not only on comorbidity in reference to index conditions, or issues associated with particular combinations of conditions, but also on an understanding of the generic, underlying commonalities of MCCs in light of the enormous heterogeneity of the population with MCCs (see [9, 10]). Examples include developing and evaluating pragmatic approaches to shared decision-making for treatment and care plans, regardless of what the specific conditions may be, or evaluating and refining electronic health records in order to enhance patient-centered care for people with MCCs [11].

Third, in addition to focusing on the effects of MCCs on the health and well-being of specific individuals with MCCs, attention should also be given to the interplay of MCCs on the health and well-being of the caregivers and family members of those individuals. Research on the social determinants of health underscores that living with MCCs is a complex and dynamic process that is likely to involve the health status of family members (see [12, 13]). This involves more than an appreciation of “caregiver strain,” as important as that issue is. As just one example of the heterogeneous connections between family members and their effect on health, an older adult caring for a spouse with MCCs may be dealing with his or her own set of MCCs; and, together, the issues surrounding their collective MCCs may affect the health and well-being of a grandchild for whom they care. A better understanding of the social and economic context of MCC, including family health issues of this kind, could serve as the foundation for a more comprehensive picture of the impact of living with MCCs.

In conclusion, we trust that all healthcare professionals involved in the management of patients with MCCs will not only find the articles in this special issue interesting and informative, but that they will also help to stimulate further research, discussion, and guidance on MCCs globally.
